# Supplementary material for: Expression Mapping and Functional Analysis of Orphan G-Protein-Coupled Receptor GPR158 in the Adult Mouse Brain Using a GPR158 Transgenic Mouse
Source: Biomolecules. 2023 Mar 5;13(3):479. doi: 10.3390/biom13030479 (PMC10046084; doi:10.3390/biom13030479)
Supplement: Supplementary file 1 [file biomolecules-13-00479-s001.zip › Figure S1.pdf]

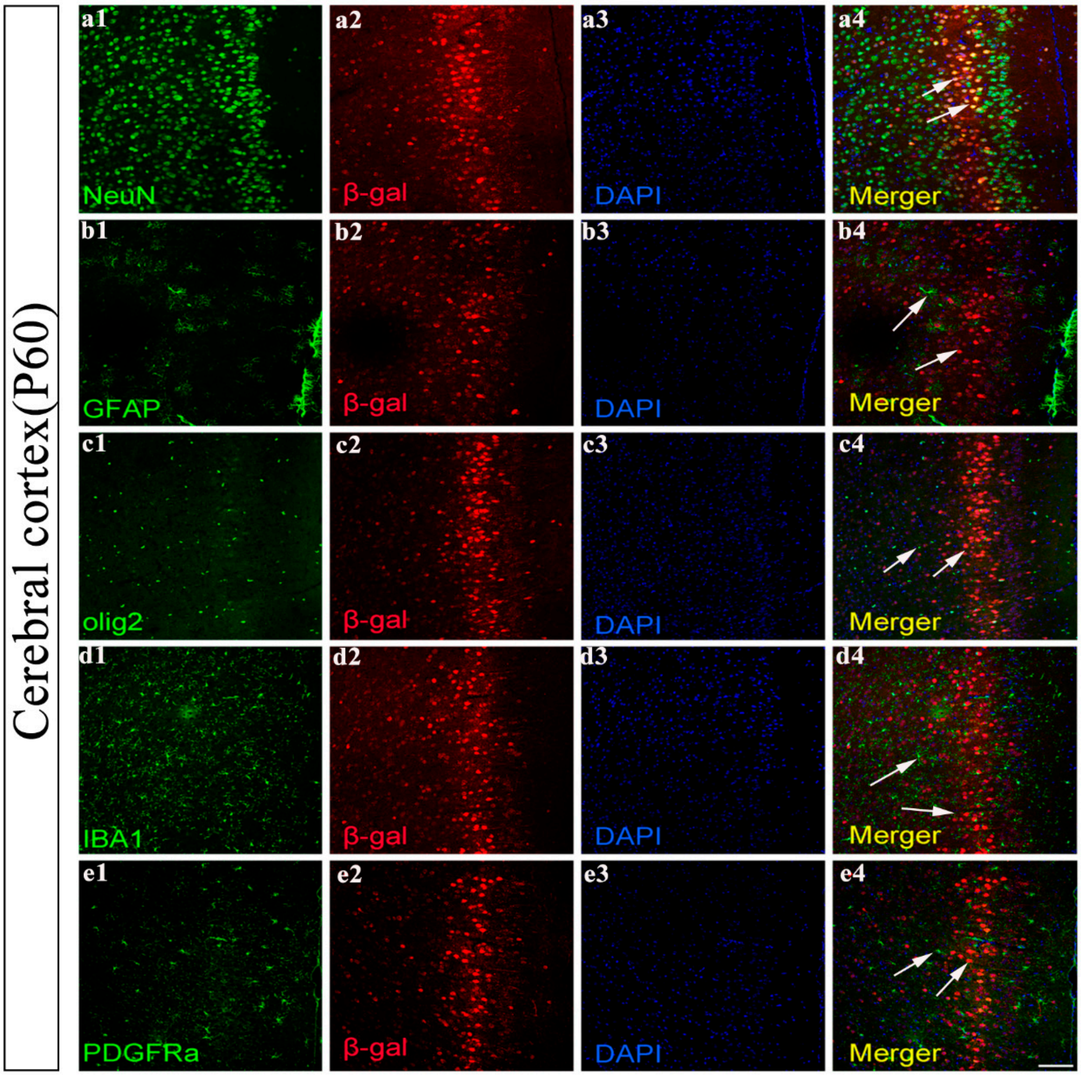

Figure S1. Expression of GPR158 in neurons of the cerebral cortex of GPR158 knockout mice at P60.

A GPR158 (red, a2) was expressed in neurons (green, a1) in the cerebral cortex of GPR158 knockout mice at P60. Neurons (green) were stained with NeuN.

B GPR158-positive cells were not detected in activated astrocytes (green) of the cerebral cortex. Activated astrocytes (green) were stained with GFAP.

C Immunofluorescence stainings on cerebral cortex showed GPR158 (red) was not expressed in oligodendrocytes. Oligodendrocytes (green) were stained with olig2.

D Immunofluorescence stainings on cerebral cortex showed GPR158 (red) was not expressed in microglia. Microglia (green) were stained with IBA1.

E GPR158 was not expressed in oligodendrocyte precursor cells(green,e1). Oligodendrocyte precursor cells (green) were stained with PDGFRa. GPR158 expression site (red) were stained with β -gal. Nuclei (blue) were stained with DAPI. Scale bars, 30 μm.
