# Supplementary material for: Expression Mapping and Functional Analysis of Orphan G-Protein-Coupled Receptor GPR158 in the Adult Mouse Brain Using a GPR158 Transgenic Mouse
Source: Biomolecules. 2023 Mar 5;13(3):479. doi: 10.3390/biom13030479 (PMC10046084; doi:10.3390/biom13030479)
Supplement: Supplementary file 1 [file biomolecules-13-00479-s001.zip › Figure S2.pdf]

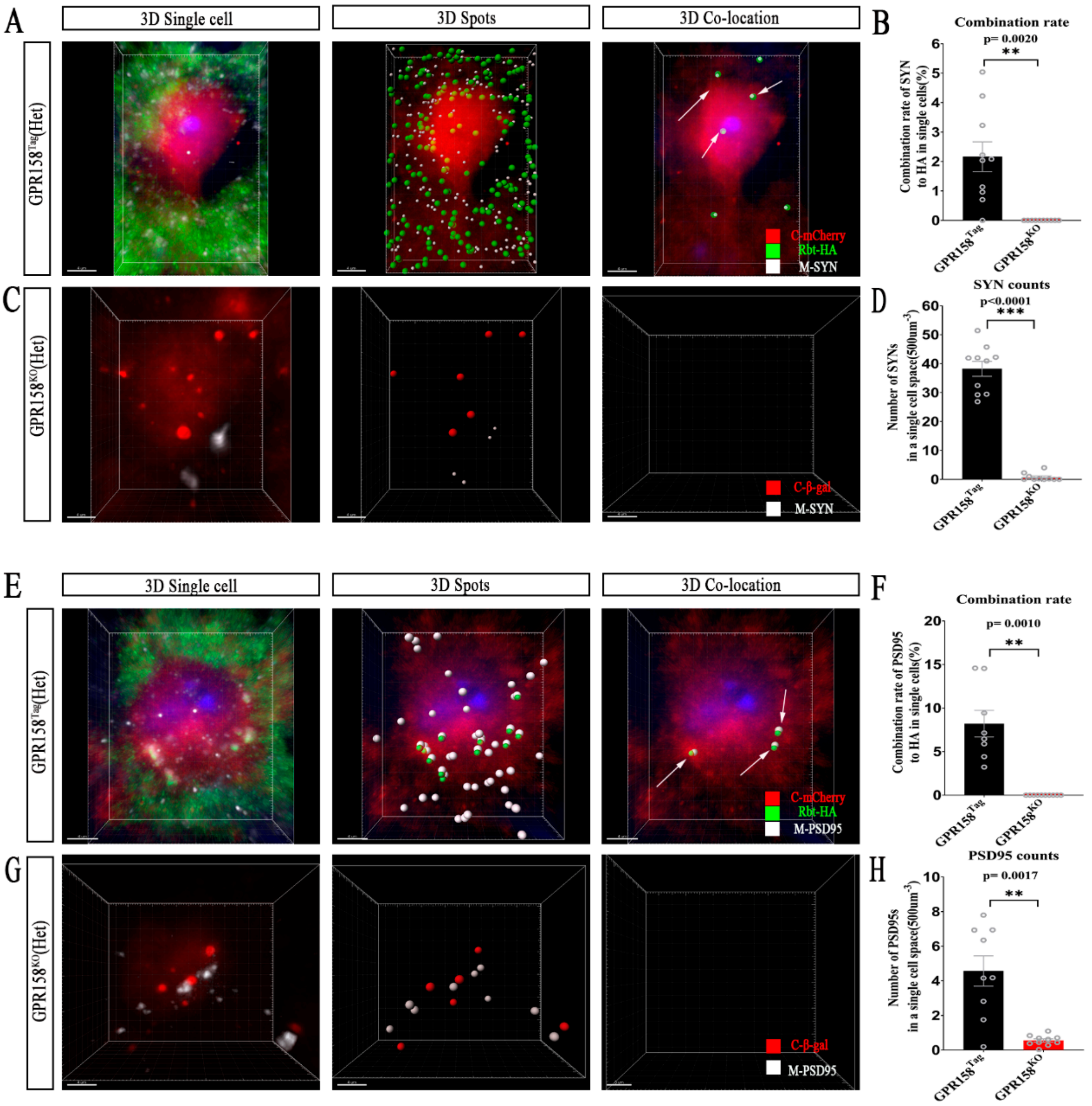

Figure S2. GPR158 knockout mice showed significant differences in the co-localization of individual neurons in the cerebral cortex in comparison to Gpr158^{Tag} mice in HA with PSD95 or SYN at P60.

A It displayed the raw immunofluorescence staining, 3D modeling and 3D co-localization of HA with SYN in the cerebral cortex of individual mCherry-positive neurons in adult Gpr158^{Tag} mice.

B It showed the scatter plot of SYN binding to HA in individual mCherry-positive cells in the cerebral cortex of Gpr158^{Tag} mice (heter) and GPR158 knockout mice (heter). mCherry^{+} cells in the cerebral cortex of Gpr158^{Tag} mice, n=10; β-gal^{+} cells in the cerebral cortex of GPR158 knockout mice, n=10; male GPR158^{Tag} mice (heter) and GPR158 knockout mice (heter) at P60; **: $P<0.01$.

C It presented the original immunofluorescence staining, 3D modelling and 3D co-localization results of β-gal with SYN in individual mCherry-positive neurons in the cerebral cortex of adult GPR158 knockout mice.

D Scatter plot of the number of SYN punctate signals around single mCherry-positive cells in the cerebral cortex of Gpr158^{Tag} mice (heter) and GPR158 knockout mice (heter). mCherry^{+} cells in the cerebral cortex of Gpr158^{Tag} mice, n=10; β-gal^{+} cells in the cerebral cortex of GPR158 knockout mice, n=10; male Gpr158^{Tag} mice (heter) and GPR158 knockout mice (heter) at P60; **: $P<0.01$.

E It illustrated the original immunofluorescence staining, 3D modelling and 3D co-localization of HA with PSD95 in the cerebral cortex of adult $Gpr158^{Tag}$ mice.

F It displayed the scatter plot of PSD95 binding to HA in individual mCherry-positive cells in the cerebral cortex of $Gpr158^{Tag}$ mice (heter) and GPR158 knockout mice (heter). $mCherry^{+}$ cells in the cerebral cortex of $Gpr158^{Tag}$ mice, n=8; β-gal^{+} cells in the cerebral cortex of GPR158 knockout mice, n=10; male $Gpr158^{Tag}$ mice (heter) and GPR158 knockout mice (heter) at P60; **: P<0.01.

G It presented the original immunofluorescence staining, 3D modeling and 3D co-localization results of β-gal with PSD95 in individual mCherry-positive neurons in the cerebral cortex of adult GPR158 knockout mice.

H It showed the scatter plot of the number of PSD95 punctate signals around single mCherry-positive cells in the cerebral cortex of $Gpr158^{Tag}$ mice (heter) and GPR158 knockout mice (heter). $mCherry^{+}$ cells in the cerebral cortex of $Gpr158^{Tag}$ mice, n=9; β-gal^{+} cells in the cerebral cortex of GPR158 knockout mice, n=10; male $Gpr158^{Tag}$ mice (heter) and GPR158 knockout mice (heter) at P60; **: P<0.01. Scale bars, 4 μm.
