# Supplementary material for: Expression Mapping and Functional Analysis of Orphan G-Protein-Coupled Receptor GPR158 in the Adult Mouse Brain Using a GPR158 Transgenic Mouse
Source: Biomolecules. 2023 Mar 5;13(3):479. doi: 10.3390/biom13030479 (PMC10046084; doi:10.3390/biom13030479)
Supplement: Supplementary file 1 [file biomolecules-13-00479-s001.zip › Figure S3.pdf]

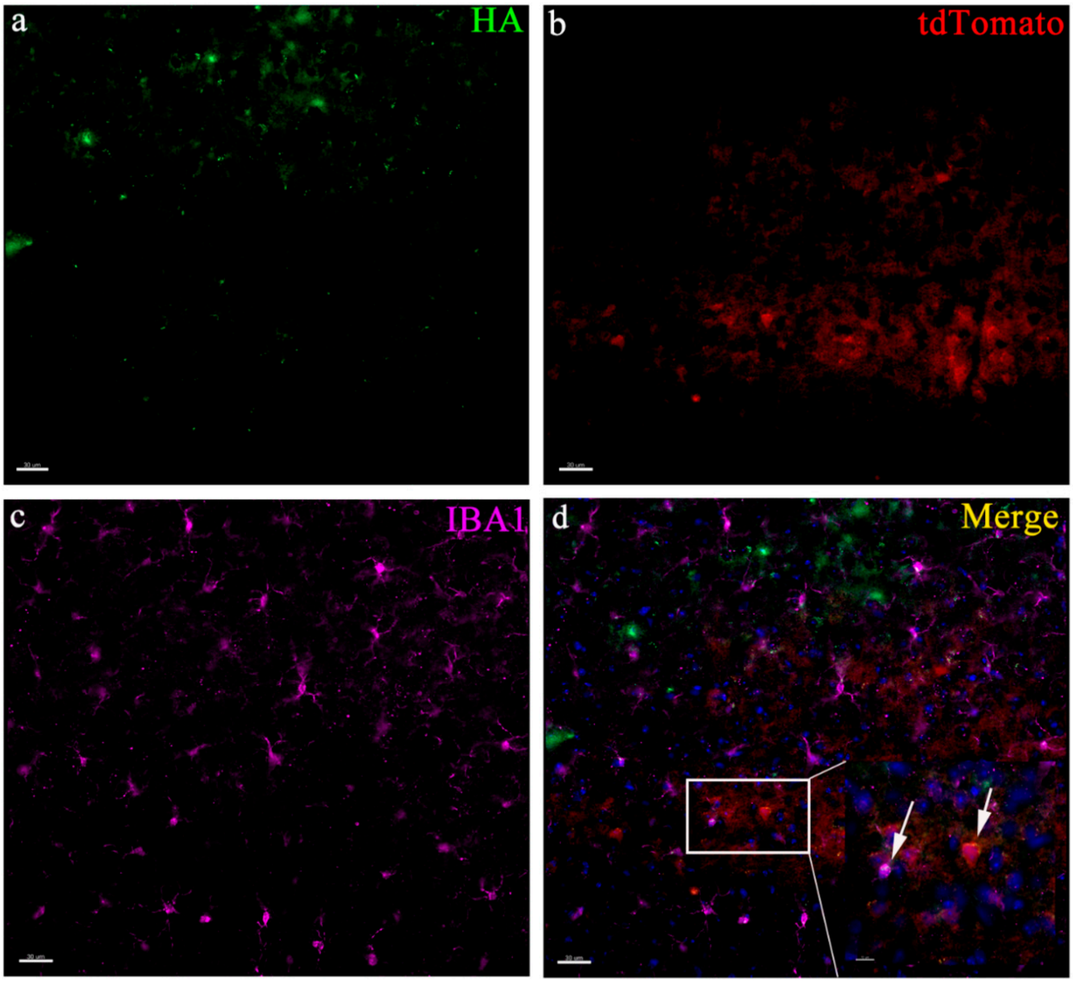

Figure S3. Microglia were not co-stained with tdTomato^{+} cells, but HA-tagged GPR158 molecules were detected in microglia in the cerebral cortex of GPR 158^{Tag} mice at P60.

A Immunofluorescence staining showing punctate signals of HA-tagged GPR158 molecules in the cerebral cortex of GPR158^{Tag} mice

B Immunofluorescence staining showing positive cells expressing GPR158 labelled by tdTomato in the cerebral cortex of GPR158^{Tag} mice

C Immunofluorescence staining showing microglia labelled by Iba1 antibody in the cerebral cortex of GPR158^{Tag} mice

D Immunofluorescence staining merge diagram and its local magnification. GPR158(red) was stained with mCherry. Nuclei (blue) were stained with DAPI. Scale bars 30 µm.
