# Supplementary material for: Expression Mapping and Functional Analysis of Orphan G-Protein-Coupled Receptor GPR158 in the Adult Mouse Brain Using a GPR158 Transgenic Mouse
Source: Biomolecules. 2023 Mar 5;13(3):479. doi: 10.3390/biom13030479 (PMC10046084; doi:10.3390/biom13030479)
Supplement: Supplementary file 1 [file biomolecules-13-00479-s001.zip › Figure S4.pdf]

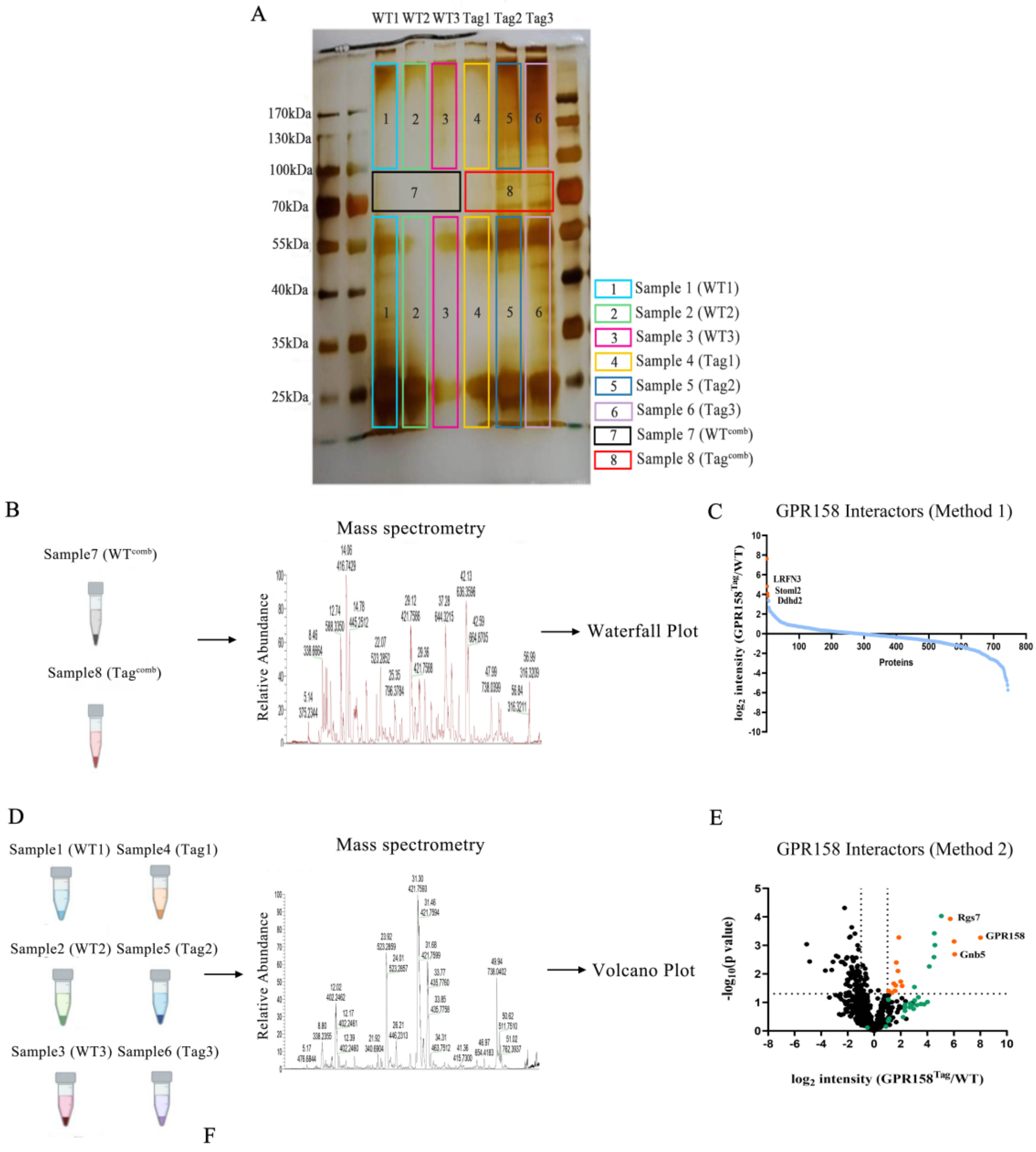


F

Proteins potentially interacting with GPR158

Method 1

Lrfn3 Srsf2 Paip1 Wdr26 Ppp2r1a
Stoml2 Rgs7 Camk2g Aldh1l1 Ipo8
Ddhd2 Eno2 Tpi1 Puf60

Method 2

Gnb5 Ppp2r2a Eva1a Immt Prmt5
Syt2 Camk2a Rgs7 Ina Mat2a
Rgs7 Try10 Gna Tn Nova2
Snap25 Stoml2 Ctps1 Igkv14-130 Anxa2
Camk2g Tpcn2 Eif3j1 IGHVDJ Nrxn1
Tfg Ppp2r1a Mrps5 Rgs6 Gbe1
Lrrc40 Gnb2 Hdlbp Lgi1 Kiaa0513
Tars Icam5

**Figure S4. Analysis of protein spectrum data on proteins that may interact with GPR158 in WT and GPR158$^{Tag}$ mice.**

A Co-immunoprecipitation (co-IP) silver staining results of Flag-tagged GPR158 in the whole brain of WT and GPR158$^{Tag}$ mice (WT mice: n=3; GPR158$^{Tag}$ mice: n=3). The bands were cut into 8 samples and sent together for protein spectrum.

B Schematic diagram of co-IP for mass spectrometry identification of candidate GPR158 interacting proteins (Method 1).

C Waterfall diagram made according to protein spectrum data of Sample 7 (WT$^{comb}$) and Sample 8 (Tag$^{comb}$) was used to screen the candidate GPR158 interacting protein (Method 1).

D Schematic diagram of co-IP for mass spectrometry identification of candidate GPR158 interacting proteins (Method 2).

E Volcano plots of GPR158 candidate interacting proteins were quantified by label-free quantification. A two-sided Student's t-test was used in the volcano plot. Wild-type mouse samples (Samples 1-3, n=3, male) were used as negative controls. GPR158$^{Tag}$ mouse samples (Samples 4-6, n=3, male) were used as the experimental group (Method 2). Significantly changed proteins were highlighted in red ($p < 0.05$ and >2-fold difference in intensity). GPR158 candidate interacting proteins that were present only in GPR158$^{Tag}$ mice but not in WT mice were labeled in green.

F The final list of GPR158 candidate interacting proteins from analytical Methods 1 and 2.
